# Artificial Intelligence Predictive Model for Hormone Therapy Use in Prostate Cancer

**DOI:** 10.21203/rs.3.rs-2790858/v1

**Published:** 2023-04-21

**Authors:** Daniel E Spratt, Siyi Tang, Yilun Sun, Huei-Chung Huang, Emmalyn Chen, Osama Mohamad, Andrew J Armstrong, Jonathan D Tward, Paul L Nguyen, Joshua M Lang, Jingbin Zhang, Akinori Mitani, Jeffry P Simko, Sandy DeVries, Douwe van der Wal, Hans Pinckaers, Jedidiah M Monson, Holly A Campbell, James Wallace, Michelle J Ferguson, Jean-Paul Bahary, Edward M Schaeffer, NRG Prostate Cancer AI Consortium, Howard M Sandler, Phuoc T Tran, Joseph P Rodgers, Andre Esteva, Rikiya Yamashita, Felix Y Feng

**Affiliations:** Case Western Reserve University; Stanford University; Case Western Reserve University; Artera, Inc.; Artera, Inc.; University of California San Francisco; Duke Cancer Institute Center for Prostate and Urologic Cancer; Huntsman Cancer Institute, University of Utah; Dana-Farber/Brigham Cancer Center; University of Wisconsin; Artera, Inc.; Artera, Inc.; University of California San Francisco; Artera, Inc.; Artera, Inc.; Saint Agnes Medical Center; Saint John Regional Hospital; University of Chicago Medicine Medical Group; Allan Blair Cancer Centre; CHUM - Centre Hospitalier de l’Universite de Montreal; Northwestern University Feinberg School of Medicine; NRG Oncology; Cedars-Sinai Medical Center; University of Maryland School of Medicine; NRG Oncology; Artera, Inc.; Artera, Inc.; University of California San Francisco

**Keywords:** Prostate cancer, predictive biomarker, digital pathology, AI, deep learning, phase III clinical trials

## Abstract

**Background:**

Androgen deprivation therapy (ADT) with radiotherapy can benefit patients with localized prostate cancer. However, ADT can negatively impact quality of life and there remain no validated predictive models to guide its use.

**Methods:**

Digital pathology image and clinical data from pre-treatment prostate tissue from 5,727 patients enrolled on five phase III randomized trials treated with radiotherapy +/− ADT were used to develop and validate an artificial intelligence (AI)-derived predictive model to assess ADT benefit with the primary endpoint of distant metastasis. After the model was locked, validation was performed on NRG/RTOG 9408 (n = 1,594) that randomized men to radiotherapy +/− 4 months of ADT. Fine-Gray regression and restricted mean survival times were used to assess the interaction between treatment and predictive model and within predictive model positive and negative subgroup treatment effects.

**Results:**

In the NRG/RTOG 9408 validation cohort (14.9 years of median follow-up), ADT significantly improved time to distant metastasis (subdistribution hazard ratio [sHR] = 0.64, 95%CI [0.45–0.90], p = 0.01). The predictive model-treatment interaction was significant (p-interaction = 0.01). In predictive model positive patients (n = 543, 34%), ADT significantly reduced the risk of distant metastasis compared to radiotherapy alone (sHR = 0.34, 95%CI [0.19–0.63], p < 0.001). There were no significant differences between treatment arms in the predictive model negative subgroup (n = 1,051, 66%; sHR = 0.92, 95%CI [0.59–1.43], p = 0.71).

**Conclusions:**

Our data, derived and validated from completed randomized phase III trials, show that an AI-based predictive model was able to identify prostate cancer patients, with predominately intermediate-risk disease, who are likely to benefit from short-term ADT.

## Introduction

Radiotherapy is a common form of treatment administered with curative intent, for localized prostate cancer. Trials conducted since the 1980s consistently demonstrate an improvement in oncologic outcomes when androgen deprivation therapy (ADT) is added to radiotherapy^1–5^. However, ADT has well-documented toxicity, including hot flashes, declines in libido and erectile function, loss of muscle mass, increase in body fat, osteoporosis, and potential deleterious effects on cardiac and brain health^6^.

While consistent oncologic benefits of ADT have been demonstrated, the majority of men with localized prostate cancer treated with radiotherapy alone without ADT never develop distant metastasis^5, 7–11^. Unfortunately, there remains no predictive biomarkers to identify which men specifically derive benefit from ADT with radiotherapy, and thus current guidelines recommend the use of ADT based on prognostic National Comprehensive Cancer Network (NCCN) risk groups or other methods of prognostication^12^. Gleason grading has modest prognostic ability and a plethora of tissue-based gene expression, serum, and imaging biomarkers have also been developed. While some have demonstrated improvements in prognostication^13^, none have been shown to function as predictive biomarkers for ADT use with randomized trial validation. Thus, there is a large unmet need to guide the individualized use of ADT with radiotherapy for men with localized prostate cancer.

Digital pathology has been used for years as a method to archive, visualize, and share histopathology images^14^. More recently, there has been growing interest in leveraging artificial intelligence (AI) to assist in the diagnosis and grading of prostate cancer^15–17^. Fundamentally, these efforts restrict AI to predict human interpretable and defined features (i.e. Gleason score). In a recent study, a multi-modal AI (MMAI) system leveraging digital histopathology and clinical data from five NRG Oncology phase III clinical trials, termed the MMAI Prostate Prognostic Model, was used to develop and validate prognostic models that consistently outperformed NCCN risk groups in localized prostate cancer^18^. In this study, we extend this approach by adapting MMAI Prostate Prognostic Model to develop a predictive model, based on “deep learning” that has the potential to be used to identify men who will benefit from ADT.

In this report, we used extant data from four NRG Oncology North American phase III randomized trials, i.e., NRG 9202, 9413, 9910, and 0126, with long term follow-up data, including pathology images. Data from these trials were acquired and digitized and used to train a predictive AI model for the identification of men with localized prostate cancer that were likely to derive differential benefit from the addition of ADT to radiotherapy. This predictive model for differential benefit from ADT was then validated using data from NRG/RTOG 9408, a clinical trial which randomized men to treatment with radiotherapy plus or minus 4 months of ADT; this trial consisted mostly of men with intermediate-risk prostate cancer, defined as Gleason score of 7 or a Gleason score of 6 or less with a PSA 10–20 ng/mL or a clinical stage T2b and not high-risk (**Clinical Risk Group Defined by NCCN Guidelines Prostate Cancer V.1.2022 in Supplementary Appendix**)^7–11^.

## Methods

### Ancillary Project Details and Trial

NRG Oncology randomized phase III trials conducted in men with localized non-metastatic prostate cancer that enrolled at least a subset of patients with intermediate-risk disease, included treatment with radiotherapy alone or with ADT, had long-term follow-up defined as a median follow-up greater than 8 years, and had stored histopathology slides in the NRG Oncology Biospecimen Bank were eligible for inclusion. Trials testing the use of chemotherapy were excluded. Data from five prospective phase III randomized trials (NRG/RTOG 9202, 9413, 9910, 0126, and 9408) were identified and used for the development and validation of a predictive model for the escalation of hormone therapy in patients with localized prostate cancer^7–11^. NRG/RTOG 9408 was used as the validation cohort in this study as it represents one of the largest phase III clinical trials evaluating patients who received radiotherapy with or without 4 months of ADT. All image data from the remaining trials were used for the image feature extraction model, and full image, clinical and outcome data from NRG/RTOG 9910 and 0126 were used for downstream predictive model development.

Details of the eligibility criteria, including the case definitions for intermediate- and high-risk disease, for each trial and the development and validation cohorts can be found in **Tables S1 and S2 in Supplementary Appendix**. Briefly, NRG/RTOG 9202 enrolled men with intermediate- and high-risk prostate cancer, and randomized patients to radiotherapy with 4 vs 28 months of ADT. NRG/RTOG 9413 enrolled men with intermediate- and high-risk prostate cancer and was a 2×2 factorial trial with randomizations to 4 months of ADT sequencing and use of pelvic nodal radiotherapy. NRG/RTOG 9910 randomized men with intermediate-risk prostate cancer to radiotherapy with 16 weeks of ADT or with 36 weeks of ADT. NRG/RTOG 0126 randomized intermediate-risk patients to lower vs higher doses of radiotherapy without ADT. NRG/RTOG 9408 randomized men with low-, intermediate-, and high-risk prostate cancer to radiotherapy with or without 4 months of ADT. Trials that included the use of ADT consisted of combined androgen blockade with an LHRH agonist and an anti-androgen. Short-term ADT was defined as 4 months of ADT (and the 36 weeks of ADT in RTOG 9910 given no difference in outcomes), and long-term ADT was solely used in the experimental arm of NRG/RTOG 9202 of 28 months.

### Objective and endpoints

The primary objective was to develop and validate an AI-based predictive model that could identify differential benefit from the addition of short-term ADT to radiotherapy in localized prostate cancer. The primary endpoint was time to distant metastasis, measured from time of randomization until development of distant metastasis or last follow-up. The secondary objective was to evaluate the predictive model on a secondary endpoint, prostate cancer-specific mortality (defined in the present study as death in the setting of distant metastasis). Metastasis-free survival (MFS, distant metastasis or death from any cause) and overall survival (OS) were evaluated as exploratory endpoints.

### Histopathology image acquisition

Unannotated hematoxylin and eosin (H&E)-stained histopathology slides in patients with localized prostate cancer from the NRG Oncology Biospecimen Bank were independently digitized without access to clinical outcomes data. The slides were digitized using a Leica Biosystems Aperio AT2 digital pathology scanner at a 20x magnification level.

### Image feature extraction model development

The first component of model development was image feature extraction, which was trained on images only to recognize defining tissue features and did not evaluate any clinical variables or outcomes. For each patient the tissue across all available digital slides were divided into 256 × 256-pixel patches. A Resnet-50 feature extraction model was trained on image patches using self-supervised learning (SSL)^19^. We employed the MoCo-v2 training protocol without access to any clinical or outcomes data^20^. Over 2.5 million tissue patches across the four trials (NRG/RTOG 9202, 9413, 9910, and 0126) were fed through the model 200 times to train this model.

### Downstream multimodal predictive model development

The second component of model development was downstream multimodal predictive model development, which evaluated the association between all features–clinical and image–with clinical outcomes, and included patients from NRG/RTOG 9910 and 0126. Since the other two trials (NRG/RTOG 9202 and 9413) are predominantly high-risk, these two were excluded from downstream predictive model development to ensure that the development set had a similar patient population as the target population for the predictive model (i.e. intermediate-risk prostate cancer). Both NRG/RTOG 9910 and 0126 were included in downstream multimodal predictive model development since each contribute to one treatment type of interest (radiotherapy + short-term ADT vs radiotherapy only, respectively; see **Methods for Multimodal Deep Learning Model Development Section in Supplementary Appendix**). Then, the model development cohort was further stratified by treatment type and randomly split into training (60%) and tuning (40%) sets for model training and hyperparameter tuning, respectively^21,22^. Clinical data, image data, and treatment types were used as inputs to a multimodal predictive model architecture (**Figure S1A in Supplementary Appendix**). The treatment type was used only for model development; treatment type was not required for model score generation on the locked model. The image and clinical data were pre-processed as specified in the **Methods for Multimodal Deep Learning Model Development Section in Supplementary Appendix**.

The multimodal predictive model optimized the difference in the magnitude of ADT benefit, outputting a continuous score ‘delta’ (**Figure S1A in Supplementary Appendix**). The 67th percentile of the delta scores in the development set was selected as the cutoff threshold as it maximized the difference between predictive model subgroup treatment effects in the tuning set and would result in reasonably sized predictive model subgroups for clinical utility. Patients with a delta score greater than the cutoff are classified as predictive model positive and those below the cutoff as predictive model negative (**Figure S1B in Supplementary Appendix**). Model development was performed using Python programming language (Python Software Foundation. Python Language Reference, version 3.8.12. Available at http://www.python.org). After the model was locked, it was provided to independent biostatisticians (HCH and JZ) to perform clinical validation of the model in NRG/RTOG 9408.

### Statistical Analysis

The NRG/RTOG 9408 validation cohort characteristics by predictive model status (positive or negative) were reported and compared using chi-square test or Fisher’s exact test in the presence of low cell counts for categorical variables, and Wilcoxon rank-sum test for continuous variables. Time to event was analyzed using the cumulative incidence function; for distant metastasis and prostate cancer-specific mortality, death without the corresponding event was treated as a competing risk. Fine and Gray regression was also performed to estimate the subdistribution hazard ratio (sHR) and 95% confidence interval (CI) for the short-term ADT treatment effect for distant metastasis and prostate cancer-specific mortality^23^. A test for predictive model-treatment interaction was performed to evaluate this predictive model. Treatment effects of the predictive model positive and negative subgroups were similarly assessed as the overall validation cohort to measure the relative treatment effect between arms. Fifteen-year restricted mean survival times were reported to provide alternative estimates given non-proportional hazards were observed^10^.

Exploratory subgroup analyses were performed where the primary analysis was reanalyzed within NCCN low- and intermediate-risk patients. Due to stage and Gleason score migration, low-risk patients from NRG/RTOG 9408 are more similar to contemporary intermediate-risk patients and were included in the subgroup analyses. Statistical analyses were performed using R, version 3.5.1 (R Foundation for Statistical Computing, Vienna, Austria). No multiplicity adjustments for the secondary and exploratory endpoints were defined. Therefore, only point estimates and 95% confidence intervals are provided. The confidence intervals have not been adjusted for multiple comparisons and should not be used to infer definitive treatment effects. Differences in percentages may not add up due to rounding.

## Results

### Patient and Model Characteristics

Of the 7,752 eligible patients enrolled on the five phase III randomized trials, 6,020 (77.7%) patients had available slides at the NRG Biospecimen Bank. Of these patients, 5,727 (95.1%) had available pre-treatment prostate slides. Pre-treatment slides were not available for 285 patients and 8 patients had insufficient tissue. Additionally, 39 patients with transurethral resection of the prostate samples were further excluded from the validation cohort (NRG/RTOG 9408). Details regarding the representativeness of the trial patients are provided in **Table S3 in Supplementary Appendix**^24^.

The development cohort for the downstream predictive model for differential benefit from ADT had 2,024 patients with a median follow-up of 10.6 years, and 1,050 (52%) patients received radiotherapy alone and 974 (48%) patients received radiotherapy with short-term ADT (**Table S2 and Table S4 in Supplementary Appendix**). The median PSA was 9 ng/mL (interquartile range [IQR], 6–13), 87% had intermediate-risk disease, and the median age was 71 years (IQR, 65–74). The final locked model was comprised primarily of histopathology features (Gleason score and imaging features), contributing to more than 86% of model prediction (**Figure S2 in Supplementary Appendix**). While histopathology features provide a large contribution, the multi-modal AI architecture utilizes deep learning and also captures interaction effects, with the model benefitting from learning of all features.

**Figure 1:**
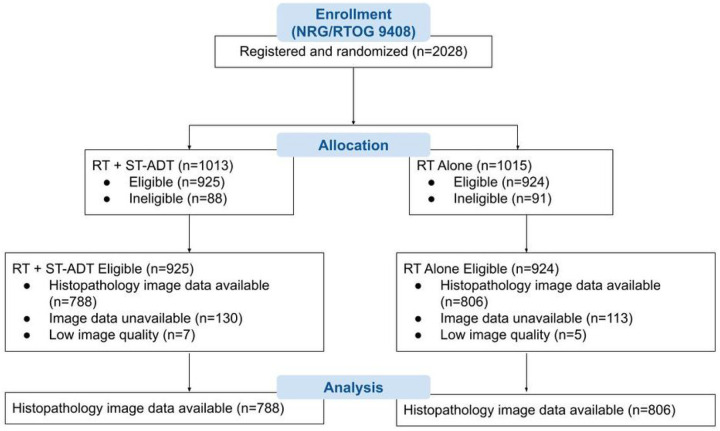
CONSORT flow diagram for NRG/RTOG 9408 (validation set). ST-ADT = short-term androgen-deprivation therapy; RT = radiotherapy.

The validation set (NRG/RTOG 9408) consisted of 1,594 patients with a median follow-up of 14.9 years, with the arms reasonably balanced in size (RT alone = 806 patients, and RT plus short-term ADT = 788 patients; Fig. 1 and Table 1). The median PSA was 8 ng/mL (IQR, 6–12), 56% had intermediate-risk disease, and the median age was 71 years (IQR, 66–74). To evaluate representativeness of the overall trial cohort, baseline characteristics between trial arms, evaluable cohort and original eligible cohorts for NRG/RTOG 9408 trial were outlined in Table 1. In the validation set, 543 patients (34%) were classified as predictive model positive (predicted to benefit most from short-term ADT), and 1,051 patients (66%) were predictive model negative (predicted to derive lesser or no benefit from short-term ADT). Baseline characteristics were generally well-matched between predictive model positive and negative patients, except Gleason score where 24% predictive model positive patients versus 30% predictive model negative patients had a Gleason score 7 (**Table S5 in Supplementary Appendix**).

Short-term *ADT Predictive Model*

**Figure 2:**
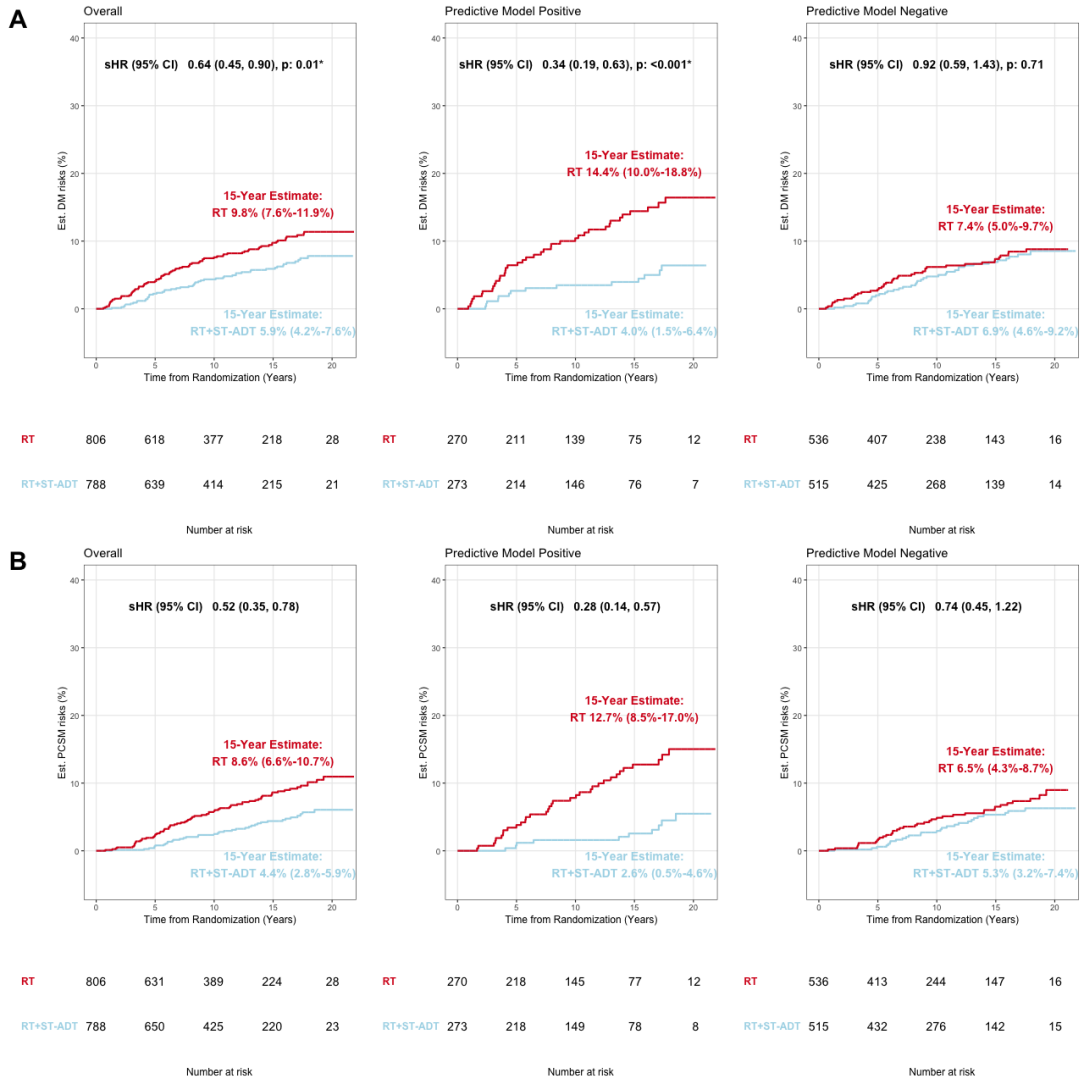
Cumulative incidence in the validation cohort, NRG/RTOG 9408, histopathology-imaged patients by AI-predictive model subgroups for A) distant metastasis and B) prostate cancer-specific mortality. Est. = estimated; DM = distant metastasis; sHR = subdistribution hazard ratio; CI = confidence interval; p = p-value; RT = radiotherapy; ST-ADT = short-term androgen-deprivation therapy; PCSM = prostate cancer-specific mortality.

**Figure 3:**
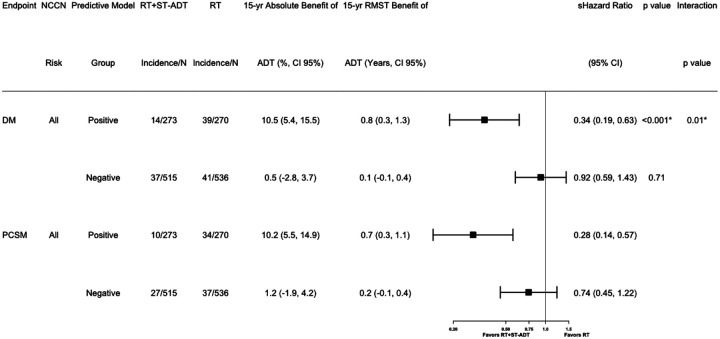
Forest plots for all endpoints in positive and negative predictive model groups of NRG/RTOG 9408 (validation set) for all patients. RT = radiation therapy; ST-ADT = short-term androgen-deprivation therapy; yr = year; RMST = restricted mean survival time; sHR = subdistribution hazard ratio; CI = confidence interval; N = number of patients; DM = distant metastasis; PCSM = prostate cancer-specific mortality.

In the overall validation cohort, the short-term-ADT group had 15-year distant metastasis estimates of 5.9% (95%CI 4.2%−7.6%) compared to the 15-year distant metastasis estimates in the radiotherapy alone group of 9.8% (95%CI 7.6%−11.9%); sHR 0.64 (95%CI [0.45–0.90], p = 0.01, Fig. 2A). Applying the locked AI-derived model to the validation set, patients identified as predictive model positive, addition of short-term ADT had a 15-year distant metastasis estimates of 4.0% (95% CI 1.5%−6.4%) compared to radiotherapy alone with a 15-year distant metastasis estimates of 14.4% (95% CI 10.0%−18.8%); sHR 0.34 (95%CI [0.19–0.63], p < 0.001, Fig. 2A). In contrast, for the patients identified as predictive model negative, two treatment groups had 15-year distant metastasis estimates of 6.9% (95% CI 4.6%−9.2%) and 7.4% (95% CI 5.0%−9.7%), respectively; sHR 0.92 (95%CI [0.59–1.43], p = 0.71, Fig. 2A). The interaction between treatment and predictive model for time to distant metastasis was analyzed with a p-value of 0.01, Fig. 3A. The absolute benefit of short-term ADT, measured as the difference in distant metastasis between treatment arms at 15 years after randomization, was 10.5 percentage points (95%CI 5.4%−15.5%, i.e., 4.0% vs 14.4% event estimates; Fig. 2A and 3) in predictive model positive patients. In contrast, in patients with predictive model negative disease there was a 0.5 percentage point (95%CI −2.8%−3.7%, 6.9% vs 7.4%) reduction in 15-year distant metastasis risk from the addition of ADT. Similarly, the short-term ADT benefit on distant metastasis measured by the restricted mean survival times at 15 years was 0.8 years (95% CI 0.3–1.3) in predictive model positive patients and 0.1 years (95% CI −0.1–0.4) in predictive model negative patients.

The secondary endpoint prostate cancer-specific mortality was also assessed (Fig. 2B and 3). In the overall validation cohort, the short-term ADT group had a 15-year event estimates of 4.4% (95% CI 2.8%−5.9%) while the radiotherapy alone group had a 15-year event estimates of 8.6% (95% CI 6.6 %−10.7%); sHR 0.52 (95%CI [0.35–0.78], Fig. 2B). Predictive model positive patients had a 15-year prostate cancer-specific mortality estimates of 2.6% (95% CI 0.5%−4.6%) if randomized to additional short-term ADT and 12.7% (95% CI 8.5%−17.0%) if randomized to radiotherapy only; sHR 0.28 (95%CI 0.14–0.57). In contrast, for predictive model negative patients, 15-year event estimates were 5.3% (95% CI 3.2%−7.4%) for additional ADT and 6.5% (95% CI 4.3%−8.7%) for RT alone; sHR 0.74 (95%CI 0.45–1.22, Fig. 2B). Absolute differences in prostate cancer-specific mortality risks at 15 years were 10.2 percentage points (event estimates: 2.6% vs 12.7%) vs 1.2 percentage points (event estimates: 5.3% vs 6.5%) in predictive model positive and negative subgroups, respectively. The short-term ADT benefit on prostate cancer-specific mortality restricted mean survival times at 15 years was 0.7 years (95% CI 0.3–1.1) in predictive model positive patients and 0.2 years (95% CI −0.1–0.4) in predictive model negative patients (Fig. 3).

On exploratory subset analysis, when restricting the analyses to solely patients with low- and intermediate-risk disease the results remained similar (**Figure S3 in Supplementary Appendix**).

We did not observe differential treatment benefits between predictive model subgroups on the exploratory endpoints, MFS and OS (p-interaction = 0.31 and 0.23, respectively; **Figure S4 in Supplementary Appendix**). The predictive model effects on distant metastasis and prostate cancer-specific mortality were evaluated within each treatment arm (**Table S6 in Supplementary Appendix**). For distant metastasis, within the RT alone arm, the predictive model positive vs negative subgroup sHR was 1.93 (95% CI 1.24–2.98), whereas within the RT + short-term ADT arm, the predictive model sHR was 0.72 (95% CI 0.39–1.34); similar results were found for prostate cancer-specific mortality as well.

## Discussion

The current standard of care for men with intermediate-risk, specifically unfavorable intermediate-risk, localized prostate cancer treated with RT is the addition of short-term ADT. Despite the improvement in outcomes in all-comers, the majority of men will not develop distant metastasis with RT alone, and many will experience side effects from ADT. Unfortunately, there are no validated predictive models to guide ADT use or duration in these men. Herein, we report our results using novel deep learning methodology and leveraging image data from over 5,000 patients on five phase III randomized trials with long-term follow-up to create and validate a predictive model to guide ADT use with RT in men with localized prostate cancer.

As a patient’s prognosis worsens (i.e., going from NCCN low- to high-risk) the recommendations to add ADT to RT strengthen. This is despite evidence that NCCN risk groups are not predictive of ADT benefit^5^. To this point, we demonstrate that among patients with positive and negative AI model predictions, the baseline PSA, T-stage, and NCCN risk group distribution, were similar; there were small differences in Gleason score. These results confirm that historical categorization of tumor aggressiveness alone is insufficient to determine which patients derive differential relative benefit from ADT.

A concern with any model is the possibility of overfitting and failure to validate. This cannot be overstated, and independent validation remains necessary to prove the performance of a model. In the specific case of predictive models, which aim to identify those patients who derive greater or lesser relative benefit, this almost always should be performed within the context of a randomized trial of the treatment of interest to avoid confounding and bias between arms. Herein, we intentionally selected NRG/RTOG 9408, as it remains the largest published trial of radiotherapy with or without short-term ADT with very long-term follow-up. While there was clear benefit of ADT in unselected patients in this trial, the majority of patients enrolled had no demonstrable benefit. Our results indicate that over 60% of the intermediate-risk patients enrolled on NRG/RTOG 9408 could be spared the morbidity and costs of ADT.

The primary endpoint of time to distant metastasis was specifically selected to train the short-term ADT predictive model. Other endpoints, such as biochemical recurrence, metastasis-free survival (MFS), and OS all have clinical relevance, but in the context of localized prostate cancer model development have notable limitations. ADT inhibits PSA production, and thus ADT is expected to delay biochemical recurrence irrespective of subgroup. Furthermore, the majority of biochemical recurrence events do not result in metastasis or death^25^. Therefore, it is a suboptimal endpoint for model training to determine intrinsic tumor-specific benefit from ADT. MFS and OS are important endpoints for determining the net effect of a given therapy and are the gold-standard for clinical trial design as they also capture death from competing causes. However, they are suboptimal endpoints for development of prostate cancer-specific predictive models for localized disease. This is because 78% of deaths in the validation cohort were not from prostate cancer, and only 12% of events in the MFS endpoint were from metastatic events. Thus, the strongest prediction models for MFS and OS would be driven by variables associated with death from non-prostate cancer causes (i.e., comorbid conditions). Importantly, despite the model being trained for distant metastasis, it showed a clear differential impact of ADT by predictive model status for prostate cancer-specific mortality, a cancer-driven endpoint.

As with any model, generalizability is critical. Concerns have been raised from AI models derived from a limited number of centers and in cohorts with limited diversity. Due to the limitations of the available data, we were unable to fully account for the potential confounding effect of factors impacting various aspects of health (e.g., socioeconomic status). Fortunately, NRG/RTOG enrolls patients from over 500 centers across primarily the USA and Canada from academic, community, and Veterans Affairs centers, and 20% of the 1,594 patients in the validation cohort were African American, which is higher than the proportion of African American men (15.6%) diagnosed with localized prostate cancer in the United States^26^. This important real-world diversity strengthens the generalizability of our findings. However, this study was underpowered to further assess the predictive performance of the model for African American men and future studies are needed for evaluation.

The study has limitations. Similar to other prognostic and predictive models in active clinical use, our short-term ADT predictive model was not developed and validated as part of a *de novo* prospective model dedicated trial. This approach is supported by Simon et al, and use of a randomized trial of RT with or without ADT strengthens the credibility and level of evidence of our work^27^. During the era of conduct and follow-up of this trial, there was effectively no use of advanced molecular imaging. Grade migration due to changes in the Gleason grading system may also have impacted patient stratification into NCCN risk groups. However, any potential biases introduced by this are likely random and impact both trial arms, and the raw histopathology imagery would not be impacted by changes in definitions of grading over time. Information on other prognostic clinicopathologic variables, such as percentage Gleason pattern 4 or percent positive biopsy cores were not available. Thus, alternative risk-classifications schemas for exploratory analyses were not performed^28,29^.

## Conclusions

We have developed and independently validated in a completed phase III randomized trial an AI-based predictive model to guide ADT use with radiotherapy in localized prostate cancer using a novel, multimodal digital pathology AI-derived digital pathology-based platform. Using this predictive model, we showed from the trial data that the majority of intermediate-risk patients did not benefit from ADT treatment.

## Figures and Tables

**Table 1.** Patient baseline characteristics for NRG/RTOG 9408.

	NRG/RTOG 9408 Full Cohort N = 1974			NRG/RTOG 9408 Imaged Cohort N = 1594	
Characteristic	Overall N = 1974^1^	Imaged N = 1594^1^	Nol Available N = 380^1^	RT N = 806^1^	RT+ST-ADT N = 788^1^
**Aim**					
RT	990 (50.2%)	806 (50.6%)	184 (48.4%)	-	-
RT+ST-ADT	984 (49.0%)	788 (49.4%)	196 (51.0%)	-	-
**Age**					
Median (IQR)	71 (66, 74)	71 (66, 74)	70 (66, 74)	71 (66, 74)	70 (66, 74)
(Missing)	1	0	1		
**Race**					
African American	394 (20.0%)	306 (19.2%)	86 (23.2%)	150 (18.6%)	156 (19.6%)
White	1,497 (75.8%)	1,220 (76.5%)	277 (72.9%)	624 (77.4%)	596 (75.6%)
Other	80 (4.1%)	65 (4.1%)	15 (3.9%)	31 (3.8%)	34 (4.3%)
Unknown	3 (0.2%)	3 (0.2%)	0 (0.0%)	1 (0.1%)	2 (0.3%)
**KPS**					
70–80	154 (7.8%)	126 (7.9%)	28 (7.4%)	60 (7.4%)	66 (8.4%)
90–100	1,819 (92.2%)	1,468 (92.1%)	351 (92.6%)	746 (92.6%)	722 (91.6%)
(Missing)	1	0	1		
**Baseline PSA (ng/mL)**					
Median (IQR)	8 (6, 12)	8 (6, 12)	7 (5, 10)	8 (6, 12)	8 (6, 12)
<4	209 (10.6%)	145 (9.1%)	64 (16.9%)	66 (8.2%)	79 (10.0%)
4–10	1,089 (55.2%)	874 (54.6%)	215 (56.7%)	448 (55.6%)	426 (54.1%)
10–20	669 (33.9%)	570 (35.8%)	99 (26.1%)	288 (35.7%)	282 (35.8%)
>20	6 (0.3%)	5 (0.3%)	1 (0.3%)	4 (0.5%)	1 (0.1%)
(Missing)	1	0	1		
**Tumor Stage**					
T1	962 (48.8%)	775 (48.6%)	187 (49.3%)	379 (47.0%)	396 (50.3%)
T2	1.011 (51.2%)	819 (51.4%)	192 (50.7%)	427 (53.0%)	392 (49.7%)
(Missing)	1	0	1		
**Nodal Stage**					
N0	80 (4.1%)	67 (4.2%)	13 (3.4%)	33 (4.1%)	34 (4.3%)
Nx	1,893 (95.9%)	1,527 (95.8%)	366 (96.6%)	773 (95.9%)	754 (95.7%)
(Missing)	1	0	1		
**Gleason Score**					
<7	1,212 (62.9%)	969 (62.2%)	243 (65.7%)	475 (60.6%)	494 (63.9%)
7	535 (27.8%)	437 (28.1%)	96 (26.5%)	233 (29.7%)	204 (26.4%)
8–10	180 (9.3%)	151 (9.7%)	29 (7.8%)	76 (9.7%)	75 (9.7%)
(Missing)	47	37	10	22	15
**Risk Group**					
High	180 (9.3%)	151 (9.7%)	29 (7.8%)	76 (9.7%)	75 (9.7%)
Intermediate	1,071 (55.6%)	878 (56.4%)	193 (52.2%)	453 (57.8%)	425 (55.0%)
Low	676 (35.1%)	528 (33.9%)	149 (40.0%)	255 (32.5%)	273 (35.3%)
(Missing)	47	37	10	22	15

1 n(%)

Note that some percentages may not add up to a hundred percent due to rounding.

N = number of patients; RT = radiation therapy; ST-ADT = short-term androgen-deprivation therapy; IQR = interquartile range; KPS = Karnofsky performance status; PSA = prostate-specific antigen; ng/mL = nanograms per milliliter.

Karnofsky performance status scores range from 0 to 100. A higher score indicates the patient having better ability to carry out daily activities.

## Data Availability

The data published in this article will be publicly available six months from publication, through requests made to NRG Oncology at APC@nrgoncology.org.

## Model Availability Statement:

The model used in this study is proprietary and currently available to patients as part of a clinical test that may be ordered by physicians through the Artera laboratory. It is not currently available for public research use due to commercial restrictions.
